# Interpersonal communication competence, organizational climate, and academic burnout: examining indirect associations within the job demands–resources framework

**DOI:** 10.3389/fpsyg.2026.1920857

**Published:** 2026-10-29

**Authors:** Batıkan Güngör Çağıran, Yalın Kılıç, Mehmet Yaşar Kılıç

**Affiliations:** Department of Educational Management, Cyprus Aydın University, Kyrenia, Türkiye

**Keywords:** academic burnout, faculty well-being, higher education, interpersonal communication competence, organizational climate

## Abstract

**Aim:**

This study examined associations among organizational climate, interpersonal communication competence, and academic burnout among university faculty within the Job Demands–Resources framework, with particular attention to the indirect association involving communication competence.

**Methods:**

Cross-sectional survey data were obtained from 406 full-time faculty members in the Gainesville, Florida region of the United States. PLS-SEM analyses were conducted using SmartPLS 4. The model was assessed using a two-stage Mode A PLS-SEM procedure with 5,000 bootstrap resamples and percentile 95% confidence intervals. First-order organizational climate and burnout dimensions were assessed before their scores were used at the higher-order level; interpersonal communication competence was modeled reflectively using 10 items.

**Results:**

Organizational climate was positively associated with interpersonal communication competence (*β* = 0.399, *p* < 0.001). Organizational climate (*β* = −0.416, *p* < 0.001) and communication competence (*β* = −0.327, *p* < 0.001) were negatively associated with academic burnout. The indirect association was also statistically significant (*β* = −0.131, 95% CI [−0.175, −0.091]).

## 1 Introduction

Higher education systems have undergone substantial transformation due to globalization and increasing market-oriented pressures (Shin and Jung, 2014). These developments have intensified publication expectations, administrative responsibilities, and performance requirements for academics (Turner and Garvis, 2023), contributing to rising levels of academic burnout and raising concerns about both faculty well-being and institutional effectiveness (Cadena-Povea et al., 2025; Misra et al., 2021). Higher education research has also documented the recurrence of institutional crisis narratives and their influence on debates about the sector (Tight, 2024). Accordingly, academic burnout is increasingly conceptualized not only as an individual psychological condition but also as an outcome embedded in organizational and institutional structures (Maslach and Leiter, 2016b; Watts and Robertson, 2011).

The Job Demands–Resources (JD-R) model provides a robust framework for understanding burnout in academic contexts (Bakker and Demerouti, 2007; Demerouti et al., 2001; Lesener et al., 2019). The model posits that burnout develops when job demands exceed available resources, while both organizational and personal resources play critical buffering and motivational roles (Demerouti and Bakker, 2011; Naidoo-Chetty and du Plessis, 2021; Schaufeli and Taris, 2014). However, in the context of contemporary higher education, increasing structural and policy-driven demands necessitate a more integrated understanding of how institutional and individual resources interact in shaping faculty well-being.

Within this framework, organizational climate represents a key institutional resource, reflecting shared perceptions of trust, fairness, and support within the academic environment (Patterson et al., 2005; Schneider et al., 2013). Interpersonal communication competence constitutes a central personal resource in academia, where teaching, collaboration, and administrative interaction are integral to academic work (Hargie, 2011; Qeadan et al., 2025; Rubin and Martin, 1994). Interpersonal communication may facilitate access to social support, which has been associated with reduced burnout and occupational strain (Cao et al., 2024; Halbesleben, 2006).

Importantly, personal resources are embedded within organizational contexts. Supportive organizational climates foster adaptive workplace behaviors and reduce stress exposure, whereas adverse climates may be associated with greater occupational strain and burnout (Li, 2025; Wang and Kee, 2025). From an organizational perspective, organizational climate functions as an important contextual resource through which institutions can support faculty well-being.

Despite growing research on academic burnout, two important gaps remain. First, although academic burnout and its organizational correlates have been examined (Barkhuizen et al., 2014; Li, 2025), interpersonal communication competence has received comparatively less attention as a distinct personal resource in this literature. Second, further investigation is warranted into how organizational and personal resources are associated with academic burnout within the same model (Bakker and de Vries, 2021; Bakker and Demerouti, 2017; Naidoo-Chetty and du Plessis, 2021). In particular, the potential indirect associations among organizational climate, interpersonal communication competence, and academic burnout remain underexplored in higher education research.

To address these gaps, this study examines the structural relationships among organizational climate, interpersonal communication competence, and academic burnout within the JD-R framework (Bakker and Demerouti, 2017; Sabagh et al., 2018). Specifically, using a cross-sectional design, it evaluates the indirect association between organizational climate and academic burnout involving interpersonal communication competence among faculty members. By examining these theoretically informed relationships, the study provides a more nuanced understanding of how contextual workplace resources and individual competencies are associated with faculty well-being. The findings also offer practical implications for developing supportive academic environments and promoting more sustainable higher education workplaces.

## 2 Literature review

### 2.1 Interpersonal communication competence as a personal resource

Within the Job Demands–Resources (JD-R) framework, personal resources are defined as positive self-evaluations that enable individuals to exert control over their environment and maintain psychological well-being under demanding work conditions (Bakker et al., 2023; Xanthopoulou et al., 2007). In academic contexts, interpersonal communication competence, defined as the ability to interact effectively across social and professional settings (Rubin and Martin, 1994), represents an important personal resource that facilitates emotional regulation, stress management, and access to social support (Cao et al., 2024; Cohen and Wills, 1985).

Academic work is inherently relational, involving continuous interaction through teaching, supervision, research collaboration, and administrative responsibilities. In this context, interpersonal communication competence facilitates the clarification of expectations, the exchange of constructive feedback, conflict management, and the development of supportive professional relationships (Edmondson, 1999; Hargie, 2011). Conversely, difficulties in professional communication may be accompanied by reduced support and greater interpersonal strain, both of which are relevant to occupational well-being (Halbesleben, 2006; Qeadan et al., 2025).

Consistent with the JD-R model, personal resources buffer the adverse effects of job demands and strengthen individuals’ capacity to cope with work-related stressors (Bakker and Demerouti, 2017; Xanthopoulou et al., 2007). Accordingly, interpersonal communication competence is conceptualized as an important personal resource that is expected to be negatively associated with academic burnout.

### 2.2 Perceived organizational climate as a facilitating context

Within academic settings, organizational climate shapes faculty members’ workplace experiences through shared perceptions of support, trust, fairness, collaboration, and institutional practices (James et al., 2008; Patterson et al., 2005). Within the Job Demands–Resources (JD-R) framework, organizational climate is conceptualized as a key contextual resource that influences employees’ access to and utilization of personal resources (Ehrhart et al., 2014; Schneider et al., 2013).

A supportive organizational climate fosters psychological safety and constructive workplace interactions (Edmondson, 1999; Frazier et al., 2017). In higher education contexts, such conditions facilitate collaboration, problem-solving, and professional communication, thereby supporting the development and effective use of interpersonal communication competence (Hargie, 2011; Qeadan et al., 2025). In contrast, unfavorable organizational climates may constrain access to valuable resources and increase exposure to workplace stressors, thereby contributing to academic burnout (Urbina-Garcia, 2020; Wang and Kee, 2025).

Previous research further indicates that organizational climates characterized by low trust, limited collaboration, or high uncertainty are associated with a reduced capacity to manage occupational demands (Li, 2025). Conversely, supportive climates and organizational resources have been linked with more favorable academic working conditions (Li, 2025; Wang and Kee, 2025). Organizational citizenship behavior also forms part of the wider literature on constructive workplace behavior (Dewangan and Goswami, 2025; Podsakoff et al., 2000).

Accordingly, organizational climate is conceptualized as an important contextual resource that is expected to be positively associated with interpersonal communication competence and negatively associated with academic burnout.

### 2.3 Academic burnout in higher education

Academic burnout is a persistent concern in higher education, where teaching, research, publication, student supervision, and administrative responsibilities can create competing work demands (Barkhuizen et al., 2014; Cadena-Povea et al., 2025; Misra et al., 2021). Recent syntheses identify workload and psychosocial working conditions as recurring correlates of faculty burnout (Cadena-Povea et al., 2025; Urbina-Garcia, 2020). Within the Job Demands–Resources (JD-R) framework, sustained demands in the absence of sufficient resources are associated with exhaustion and disengagement from work, the two dimensions assessed in the present study (Bakker and Demerouti, 2017; Demerouti et al., 2001; Maslach and Leiter, 2016a).

Previous research has linked the experience of burnout among academics to work-related stress and organizational conditions (Sabagh et al., 2018; Velez-Cruz and Holstun, 2022; Watts and Robertson, 2011). For this reason, academic burnout is examined here in relation to organizational climate as a contextual resource and interpersonal communication competence as a personal resource. The cross-sectional design allows these associations to be estimated but cannot establish their temporal or causal direction (Bakker and Demerouti, 2017; Kline, 2016) (Figure 1).

**Figure 1:**
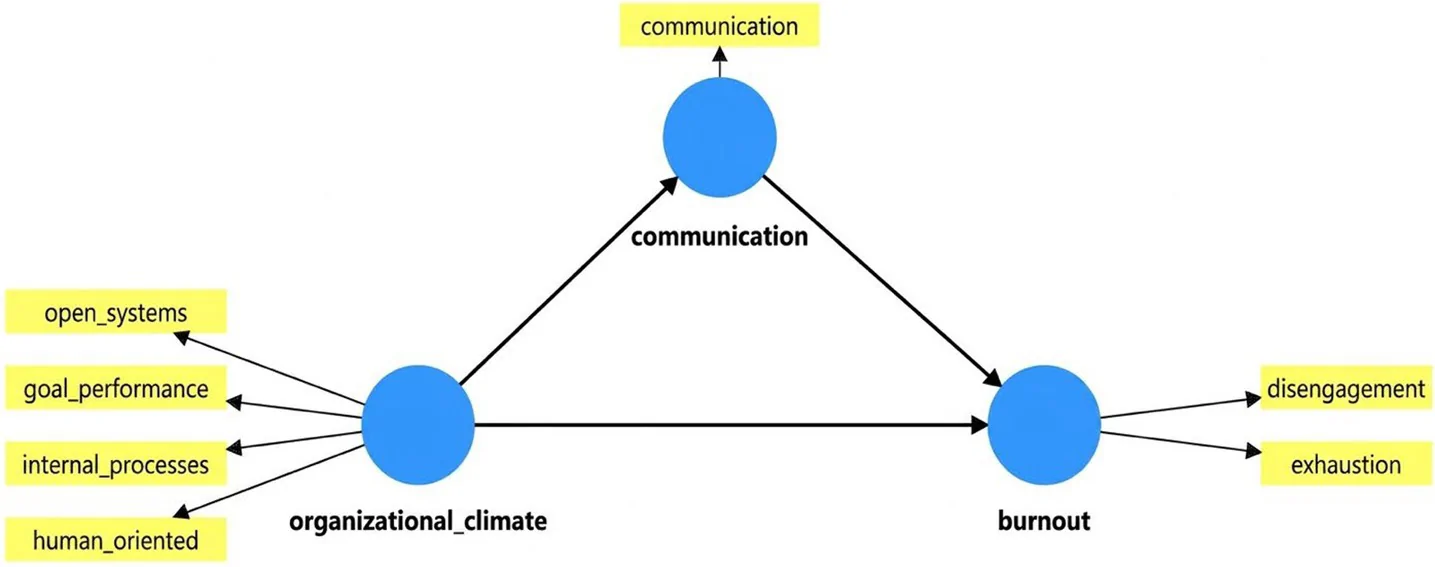
Research model.

### 2.4 Hypotheses development and research framework

Drawing on the Job Demands–Resources (JD-R) framework, this study conceptualizes organizational climate as an organizational resource and interpersonal communication competence as a personal resource (Bakker and Demerouti, 2017; Naidoo-Chetty and du Plessis, 2021). A supportive organizational climate is expected to facilitate the development and effective utilization of interpersonal communication competence (Edmondson, 1999; Frazier et al., 2017), which, in turn, is expected to be associated with lower levels of academic burnout (Cao et al., 2024). Accordingly, the study examines the structural relationships among organizational climate, interpersonal communication competence, and academic burnout, including the indirect association involving interpersonal communication competence. Based on this theoretical framework, the following hypotheses are proposed.

### 2.5 Hypotheses development

#### 2.5.1 Perceived organizational climate and interpersonal communication competence

Organizational climate reflects shared perceptions of the work environment shaped by physical, social, and managerial conditions (Patterson et al., 2005). In higher education, a climate characterized by trust, transparency, and respect fosters psychological safety and supports open communication and collaboration among faculty members (Edmondson, 1999; Frazier et al., 2017). Such an environment enables academics to develop and effectively utilize interpersonal communication competence without fear of institutional tension or professional vulnerability (Edmondson, 1999; Frazier et al., 2017). Accordingly, within the Job Demands–Resources (JD-R) framework, perceived organizational climate is conceptualized as an important contextual resource that is expected to be positively associated with interpersonal communication competence in academic and administrative settings. Based on these theoretical arguments, the following hypothesis is proposed:

*H1*: Perceived organizational climate is positively associated with academics’ interpersonal communication competence.

#### 2.5.2 Perceived organizational climate and academic burnout

A supportive and well-structured work environment represents an important organizational resource that helps employees manage workplace demands and maintain psychological well-being (Bakker and Demerouti, 2017). Within the Job Demands–Resources (JD-R) framework, a fair, transparent, and collaborative organizational climate is conceptualized as a key contextual resource that is expected to be associated with lower levels of academic burnout among faculty members (Li, 2025; Wang and Kee, 2025). By reducing ambiguity in institutional processes and promoting equitable access to organizational resources, a supportive organizational climate may be associated with lower levels of emotional exhaustion and professional depletion (Schaufeli and Taris, 2014). Based on these theoretical arguments, the following hypothesis is proposed:

*H2*: Perceived organizational climate is negatively associated with academic burnout.

#### 2.5.3 Interpersonal communication competence and academic burnout

Interpersonal communication competence is recognized as an important personal resource that enables academics to manage role conflicts, address administrative challenges, and access social support (Hargie, 2011). Within the Job Demands–Resources (JD-R) framework, interpersonal communication competence is conceptualized as a protective personal resource that helps faculty cope with job demands and is associated with lower levels of emotional exhaustion (Bakker et al., 2023; Cao et al., 2024).

By facilitating clear communication and effective social interactions within academic environments, interpersonal communication competence may be associated with lower levels of strain arising from workload and interpersonal stressors. Accordingly, interpersonal communication competence is expected to be negatively associated with academic burnout.

*H3*: Interpersonal communication competence is negatively associated with academic burnout.

#### 2.5.4 Indirect association involving interpersonal communication competence

Within the Job Demands–Resources (JD-R) framework, organizational and personal resources are conceptualized as operating jointly to support employee well-being and work-related outcomes (Bakker and Demerouti, 2017; Naidoo-Chetty and du Plessis, 2021). In higher education contexts, a supportive organizational climate is expected to be positively associated with interpersonal communication competence by fostering collaboration, trust, and psychological safety (Edmondson, 1999; Frazier et al., 2017).

Higher interpersonal communication competence, in turn, is expected to be associated with lower levels of academic burnout by supporting faculty members in managing professional demands and interpersonal challenges (Bakker et al., 2023; Hargie, 2011; Rubin and Martin, 1994). Consistent with the theoretical propositions of the JD-R framework, this study examines whether a statistically significant indirect association exists between perceived organizational climate and academic burnout through interpersonal communication competence. Based on these theoretical arguments, the following hypothesis is proposed:

*H4*: A statistically significant indirect association is expected between perceived organizational climate and academic burnout through interpersonal communication competence.

## 3 Materials and methods

### 3.1 Data collection and sample

This study employed a cross-sectional survey design. Data were collected from full-time university faculty members between September and November 2025 using a secure online survey platform. The survey was distributed through academic registries, professional networks, and institutional listservs.

Participation was voluntary, and informed consent was obtained electronically before participants accessed the survey. Responses were collected anonymously, and no personally identifiable information (PII) was recorded. The demographic characteristics of the participant sample are presented in Table 1.

**Table 1:** Demographic characteristics of study participants.

Demographic Variable	Group	Frequency	Percentage %
Gender	Male	161	39.7
	Female	245	60.3
Age	20–29 years	110	27.1
	30–39 years	129	31.8
	40–49 years	97	23.9
	50 years and above	70	17.2
Years of experience at the current university	1–5 years	151	37.2
	6–10 years	95	23.4
	11–15 years	77	19.0
	16–20 years	53	13.1
	21 years and above	30	7.4
Total working time	1–5 years	95	23.4
	6–10 years	81	20.0
	11–15 years	87	21.4
	16–20 years	79	19.5
	21 years and above	64	15.8

### 3.2 Participants

The target population comprised full-time faculty members employed at higher education institutions in the Gainesville, Florida region, a major higher education hub in the southeastern United States. A convenience sampling approach was adopted due to accessibility considerations. More than 600 faculty members were invited to participate through institutional mailing lists and professional academic networks. After screening for missing values, invalid responses, and outliers, the final sample consisted of 406 participants (*N* = 406). The sample size exceeded the recommended minimum for Partial Least Squares Structural Equation Modeling (PLS-SEM) analyses.

### 3.3 Measures

Well-established and psychometrically validated instruments were used to assess organizational climate, interpersonal communication competence, and academic burnout. The questionnaire comprised three sections corresponding to these constructs. Prior to the main study, a pilot test was conducted with 10 academic experts and faculty members to evaluate item clarity, contextual relevance, and survey completion time. Minor revisions were made based on their feedback.

Perceived organizational climate was assessed using the 25-item short form of the Organizational Climate Measure (OCM) developed by Patterson et al. (2005), covering four dimensions: human relations, internal processes, open systems, and rational goal orientation. Responses were recorded on a 4-point Likert scale ranging from 1 (*Definitely false*) to 4 (*Definitely true*), with negatively worded items reverse-coded.

Interpersonal communication competence was measured using the 10-item short form of the Interpersonal Communication Competence Scale (ICCS) developed by Rubin and Martin (1994). Responses were recorded on a 5-point Likert scale ranging from 1 (*Almost never*) to 5 (*Almost always*).

Academic burnout was measured using the 16-item Oldenburg Burnout Inventory (OLBI) developed by Demerouti et al. (2001), which comprises two dimensions: exhaustion and disengagement from work. Responses were recorded on a 5-point Likert scale ranging from 1 (Strongly disagree) to 5 (Strongly agree), with reverse-coded items recoded before analysis.

The reliability and validity of all measurement instruments were evaluated using Cronbach’s alpha, composite reliability (CR), average variance extracted (AVE), and discriminant validity indices. The corresponding results are presented in the Measurement Model section.

### 3.4 Data analysis

Prior to analysis, scored items and reverse coding were checked against the questionnaire coding. Descriptive statistics and Pearson correlations were calculated from observed scale means (Hair et al., 2019). The structural model was analyzed in SmartPLS 4 (Hair et al., 2017; Hair et al., 2022). The final item-level dataset (*N* = 406) was used to assess the two-stage PLS-SEM specification (Mode A, path-weighting scheme). First, reflective scores were derived separately for the four organizational climate dimensions (human relations, internal processes, open systems and rational goal) and the two burnout dimensions (disengagement and exhaustion). Second, the resulting dimension scores served as reflective indicators of their respective higher-order constructs, whereas interpersonal communication competence was estimated directly from its 10 scored items. No demographic covariates were entered. Inference was based on 5,000 participant-level bootstrap resamples and percentile 95% confidence intervals.

Internal consistency, composite reliability (CR), average variance extracted (AVE) and discriminant validity (HTMT) were assessed for the estimated models (Henseler et al., 2015). For transparency, the first-order construct assessments are reported separately, and Tables 2–4 refer consistently to the second-stage measurement model. An unrotated principal-components diagnostic based on all 51 scored items yielded 39.52% variance explained by the first component. This diagnostic does not, by itself, exclude common method bias. Structural collinearity was examined using the variance inflation factor (VIF; Table 5).

**Table 2:** Higher-order organizational climate: second-stage reliability, convergence and reflective loadings.

Construct	Cronbach’s α	CR	AVE	Indicator	Loading
Organizational climate	0.943	0.959	0.855	Human relations	0.925
				Internal processes	0.915
				Open systems	0.931
				Rational goal	0.928

**Table 3:** Interpersonal communication competence: reliability, convergence and item loadings.

Construct	Cronbach’s α	CR	AVE	Indicator	Loading
Communication competence	0.936	0.946	0.635	CS1	0.795
				CS2	0.811
				CS3	0.781
				CS4	0.813
				CS5	0.808
				CS6	0.795
				CS7	0.782
				CS8	0.805
				CS9	0.781
				CS10	0.797

**Table 4:** Higher-order academic burnout: second-stage reliability, convergence and reflective loadings.

Construct	Cronbach’s α	CR	AVE	Indicator	Loading
Academic burnout	0.944	0.973	0.947	Disengagement	0.973
				Exhaustion	0.973

**Table 5:** Inner-model variance inflation factors (VIF).

Structural predictor path	VIF
OC → ICC	1.000
OC → BO	1.190
ICC → BO	1.190

Structural path coefficients and the indirect association were estimated using 5,000 non-parametric case-resampling bootstrap samples, re-estimating both model stages in each sample. Two-sided approximate *p*-values were based on the estimated coefficients and bootstrap standard errors; 95% confidence intervals were percentile bootstrap intervals. Given the cross-sectional design, these results were interpreted as indirect associations rather than causal mediation.

### 3.5 Ethical considerations and approval

Data were collected using a secure online survey platform. Before participation, respondents were informed about the purpose and scope of the study and provided informed consent electronically prior to accessing the questionnaire.

The research protocol and instruments were approved by the Institutional Review Board (IRB) of Cyprus Aydın University (Approval Code: 2024/03.001; Date: March 20, 2024). The study was conducted in accordance with this approval and relevant institutional guidelines.

To ensure confidentiality, no personally identifiable information (PII) was collected. All responses were recorded anonymously and used solely for scientific purposes. The study also adhered to the ethical principles outlined in the Declaration of Helsinki.

## 4 Results

### 4.1 Descriptive statistics and correlation analysis

This subsection reports the descriptive statistics of the main constructs and examines their interrelationships using Pearson correlation analysis. Prior to the structural model analysis, data distribution and multicollinearity were assessed, and the results are presented in the table below.

Table 6 presents descriptive statistics for the observed scale means (*N* = 406). Organizational climate (*M* = 3.40, SD = 0.402), interpersonal communication competence (*M* = 3.54, SD = 0.769) and academic burnout (*M* = 2.14, SD = 0.674) were calculated using the scored questionnaire items.

**Table 6:** Descriptive statistics, normality indicators, and correlation matrix for the research variables.

Scales	Mean	SD	Skewness	Kurtosis	1	2	3
Organizational Climate	3.40	0.402	−0.186	−0.598	1		
Communication Skills	3.54	0.769	−0.630	0.200	0.393**	1	
Burnout	2.14	0.674	0.788	0.779	−0.544**	−0.492**	1

^**^ Correlation is significant at the 0.01 level (2-tailed).

Skewness ranged from −0.630 to 0.788, and excess kurtosis ranged from −0.598 to 0.779 across the three observed scale means. These descriptive indices do not establish multivariate normality and are not a prerequisite for PLS path modelling.

The Pearson correlations were positive between organizational climate and communication competence (*r* = 0.393, *p* < 0.01), and negative between organizational climate and burnout (*r* = −0.544, *p* < 0.01) and between communication competence and burnout (*r* = −0.492, *p* < 0.01). These are associations between observed scores, rather than structural path coefficients.

Table 5 shows that all structural predictor VIF values were between 1.000 and 1.190. The predictor VIF for the sole path to interpersonal communication competence was 1.000, while the two predictors of burnout each had a VIF of 1.190.

These values are below commonly used collinearity thresholds; thus, the estimates do not indicate problematic multicollinearity in the specified structural model.

### 4.2 Measurement model

Following the preliminary analyses, the measurement properties of the variables incorporated in the research were evaluated. Within this scope, the reliability and convergent validity results of the scales were examined.

In the second-stage model, organizational climate was represented reflectively by the four estimated first-order dimension scores (Table 2). Cronbach’s *α* = 0.943, CR = 0.959, and AVE = 0.855.

The standardized higher-order loadings ranged from 0.915 to 0.931. These loadings refer to the four dimension scores, not the 25 original organizational climate items. The conventional alpha coefficient computed from all 25 scored items was 0.963. The higher-order CR of 0.959 indicates substantial intercorrelation among the dimensions and therefore warrants consideration of potential overlap (Table 7).

**Table 7:** First-order reflective measurement assessment used to derive the dimension scores in stage 1.

First-order construct	Items	*α*	CR	AVE	Loading range
Human relations	8	0.895	0.916	0.578	0.745–0.788
Internal processes	4	0.813	0.877	0.642	0.774–0.818
Open systems	6	0.892	0.918	0.651	0.787–0.830
Rational goal	7	0.893	0.916	0.609	0.749–0.800
Disengagement	8	0.929	0.942	0.669	0.799–0.837
Exhaustion	8	0.927	0.940	0.664	0.799–0.838

Communication competence was estimated as a 10-indicator reflective latent construct (Table 3), rather than as a single error-free observed score. Internal consistency and convergence were α = 0.936, CR = 0.946 and AVE = 0.635.

The 10 item loadings ranged from 0.781 to 0.813. Each indicator has a non-zero measurement-error component in the reflective outer model (standardized residual variance = 1 − loading^2^).

Burnout was represented by estimated exhaustion and disengagement dimension scores (Table 4). Its second-stage reliability estimates were α = 0.944, CR = 0.973, and AVE = 0.947.

Both second-stage burnout loadings were approximately 0.973; these refer to dimension scores rather than the 16 original burnout items. The conventional alpha coefficient across all 16 scored items was 0.961; the first-order dimension assessments are given in Table 7.

To determine whether the organizational climate, communication skills, and burnout constructs incorporated in the measurement model are empirically distinct from each other, discriminant validity was evaluated via the HTMT criterion, and the generated findings are reported in Table 8.

**Table 8:** Discriminant validity results based on the heterotrait-monotrait (HTMT) ratio of correlations criterion.

Construct pair	HTMT	2.5% CI	97.5% CI
Organizational climate ↔ Communication competence	0.423	0.333	0.502
Burnout ↔ Communication competence	0.524	0.444	0.595
Burnout ↔ Organizational climate	0.579	0.505	0.648

HTMT ratios were calculated from the second-stage construct indicators; limits are 95% percentile bootstrap confidence intervals (5,000 resamples).

The second-stage HTMT ratios ranged from 0.423 to 0.579 (Table 8). Their upper 95% bootstrap limits were below 0.85, supporting discriminant validity for the specified three-construct measurement model.

### 4.3 Structural model

After assessment of both measurement stages, standardised structural associations were estimated between organizational climate, interpersonal communication competence and burnout. Interpersonal communication competence was specified reflectively by its 10 item indicators; higher-order organizational climate and burnout were specified using first-stage dimension scores. The structural model is presented in Figure 2 and Table 9.

**Figure 2:**
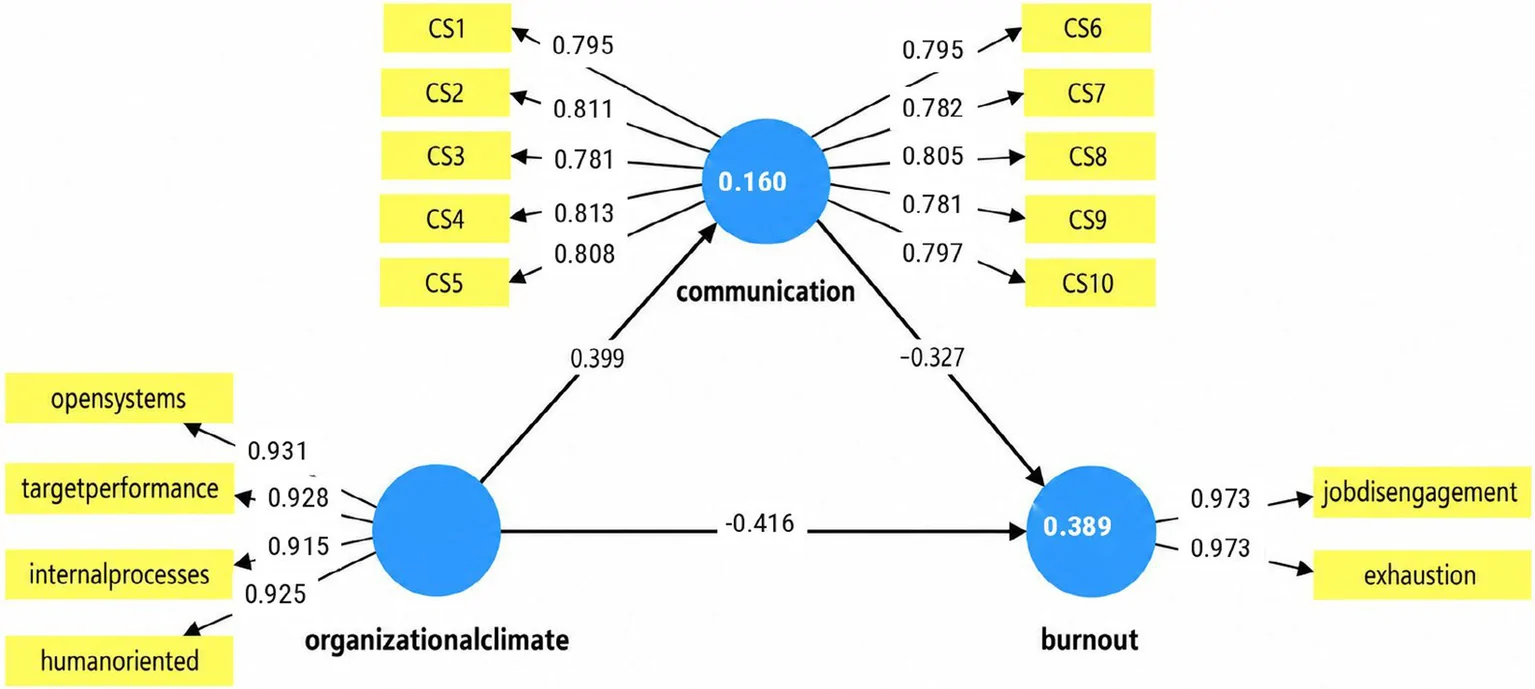
Structural model of the proposed research framework (standardized estimates).

**Table 9:** Path testing results.

Hypothesis	Path	*β*	2.5% CI	97.5% CI	*t*	*p*	Result
H1	OC → ICC	0.399	0.318	0.477	10.033	< 0.001	Supported
H2	OC → BO	−0.416	−0.493	−0.336	10.501	< 0.001	Supported
H3	ICC → BO	−0.327	−0.402	−0.252	8.536	< 0.001	Supported

*β*, standardized path coefficient; CI, 95% percentile bootstrap confidence interval (5,000 resamples); *p*, normal-approximation two-sided bootstrap *p*-value. OC, organizational climate; ICC, interpersonal communication competence; BO, burnout. No demographic covariates were included.

Table 9 shows a positive association between organizational climate and interpersonal communication competence (*β* = 0.399, 95% CI [0.318, 0.477], *p* < 0.001). Organizational climate (*β* = −0.416, 95% CI [−0.493, −0.336], *p* < 0.001) and interpersonal communication competence (*β* = −0.327, 95% CI [−0.402, −0.252], *p* < 0.001) were negatively associated with burnout. All three direct hypotheses were supported. The model accounted for 16.0% of the variance in communication competence and 38.9% of the variance in burnout.

The direction of the estimated direct paths was consistent with the theoretical expectations; the coefficients should be interpreted as cross-sectional associations rather than causal effects.

### 4.4 Analysis of indirect associations

The indirect association between organizational climate and burnout via interpersonal communication competence was evaluated using 5,000 non-parametric bootstrap samples with 95% percentile confidence intervals (Table 10).

**Table 10:** Bootstrapping results for the estimated indirect association involving interpersonal communication competence.

Hypothesis	Indirect association	*β* (indirect)	95% CI lower	95% CI upper	*t*	*p*	Result
H4	OC → ICC → BO	−0.131	−0.175	−0.091	6.136	< 0.001	Supported

*β*, standardized indirect association; CI, 95% percentile bootstrap confidence interval (5,000 resamples); *p*, normal-approximation two-sided bootstrap *p*-value. OC, organizational climate; ICC, interpersonal communication competence; BO, burnout.

The estimated indirect association was negative and statistically significant (*β* = −0.131, *t* = 6.136, *p* < 0.001, 95% CI [−0.175, −0.091]). Because the bootstrap interval excluded zero, H4 was supported. This indirect association does not establish causal mediation in a cross-sectional design.

Accordingly, the findings support Hypothesis 4. In addition, the direct association between organizational climate and academic burnout remained statistically significant, indicating that interpersonal communication competence did not fully account for the observed association. Given the cross-sectional design, these findings should be interpreted as evidence of a statistically significant indirect association rather than causal mediation.

## 5 Discussion

Grounded in the Job Demands–Resources (JD-R) framework, this study found that perceived organizational climate and interpersonal communication competence (ICC) were both negatively associated with academic burnout among university faculty members. In addition, the structural analysis identified a statistically significant indirect association between organizational climate and academic burnout involving ICC. These findings suggest that supportive organizational environments and interpersonal communication competence are jointly associated with lower levels of academic burnout.

These findings are consistent with the JD-R framework, which emphasizes the complementary roles of organizational and personal resources in helping employees manage demanding work conditions (Bakker and Demerouti, 2017; Naidoo-Chetty and du Plessis, 2021). In higher education, faculty members routinely encounter substantial teaching, research, publication, and administrative demands. The present findings suggest that the availability of both organizational and personal resources is associated with lower levels of academic burnout. Within this framework, organizational climate represents an important organizational resource, whereas interpersonal communication competence represents an important personal resource associated with faculty well-being.

The statistically significant indirect association involving interpersonal communication competence is consistent with the proposition that supportive organizational climates and communication competence are jointly associated with academic burnout. Faculty members working in supportive and collaborative environments may have greater opportunities to develop effective communication practices, build professional relationships, and access social support. These factors may, in turn, be associated with lower levels of academic burnout. Given the cross-sectional design, these findings should not be interpreted as evidence of causal mediation or temporal ordering but rather as theoretically informed indirect associations.

Overall, the findings suggest that academic burnout should be understood not only as an individual experience but also as a phenomenon associated with organizational conditions. Accordingly, institutional efforts to promote faculty well-being may benefit from strengthening supportive organizational climates while fostering interpersonal communication competence.

### 5.1 The association between organizational climate and interpersonal communication competence

The findings indicate that perceived organizational climate was positively associated with interpersonal communication competence among faculty members, supporting Hypothesis 1 (H1). Consistent with relevant organizational research (Patterson et al., 2005; Schneider et al., 2013), supportive organizational environments characterized by trust, professional autonomy, and open communication are associated with higher levels of interpersonal communication competence.

This finding is noteworthy because interpersonal communication competence is often conceptualized primarily as an individual characteristic. The present findings suggest that it is also associated with organizational conditions. Within the Job Demands–Resources (JD-R) framework, supportive organizational climates and interpersonal communication competence may therefore be viewed as complementary organizational and personal resources associated with faculty members’ ability to manage the interpersonal demands of academic work.

### 5.2 Organizational and personal resources in relation to academic burnout

The findings showed that both perceived organizational climate (H2) and interpersonal communication competence (H3) were negatively associated with academic burnout. These findings are consistent with the Job Demands–Resources (JD-R) framework (Bakker and Demerouti, 2017; Demerouti et al., 2001), which emphasizes the complementary roles of organizational and personal resources in helping employees manage demanding work conditions. Faculty members who perceived their work environments as more supportive reported lower levels of academic burnout, consistent with previous research linking organizational resources with lower burnout levels (Schaufeli and Bakker, 2004).

Interpersonal communication competence was likewise negatively associated with academic burnout. Faculty members with stronger interpersonal communication competence may be better able to manage interpersonal challenges, establish supportive professional relationships, and access social support when needed (Cao et al., 2024; Hargie, 2011). These factors may, in turn, be associated with lower levels of academic burnout.

Taken together, the findings suggest that both organizational conditions and individual competencies are associated with academic burnout. While organizational climate represents an important organizational resource, interpersonal communication competence may help faculty members make effective use of available resources while navigating the interpersonal demands of academic work.

### 5.3 Indirect association and resource caravans

Hypothesis 4 proposed that interpersonal communication competence would be involved in the indirect association between organizational climate and academic burnout. The findings supported this proposition by identifying a statistically significant indirect association involving interpersonal communication competence. This pattern is consistent with Conservation of Resources (COR) theory (Hobfoll, 1989; Hobfoll, 2011), which proposes that organizational and personal resources tend to accumulate and operate together in supporting employees’ adaptation to demanding work conditions.

Within academic settings, supportive organizational climates characterized by trust, collaboration, and open communication are associated with higher levels of interpersonal communication competence. Faculty members with stronger interpersonal communication competence may be better able to establish supportive professional relationships, access social support, and manage the interpersonal demands of academic work. Together, these organizational and personal resources are associated with lower levels of academic burnout.

The observed indirect association suggests that interpersonal communication competence represents one potential personal resource through which organizational climate and academic burnout are statistically linked. At the same time, the direct association between organizational climate and academic burnout remained statistically significant, indicating that interpersonal communication competence does not fully account for this relationship. Given the cross-sectional design, these findings should be interpreted as theoretically informed indirect associations rather than evidence of causal mediation.

Overall, the findings are consistent with the proposition that academic burnout is associated with both organizational conditions and individual resources. Future research employing longitudinal or experimental designs is needed to clarify the temporal ordering and causal processes underlying these associations.

## 6 Conclusion

This study examined academic burnout among university faculty by focusing on organizational climate and interpersonal communication competence within the Job Demands–Resources (JD-R) framework. The findings indicate that both perceived organizational climate and interpersonal communication competence were associated with lower levels of academic burnout. In addition, a statistically significant indirect association was identified between organizational climate and academic burnout involving interpersonal communication competence.

The study contributes to the higher education literature by providing further evidence that organizational climate and interpersonal communication competence are jointly associated with faculty well-being. These findings are consistent with the proposition that interpersonal communication competence represents an important personal resource within the JD-R framework. Given the cross-sectional design, the observed indirect association should not be interpreted as evidence of causal mediation but rather as a theoretically informed association requiring further investigation using longitudinal or experimental research designs.

From a practical perspective, higher education institutions may benefit from fostering supportive and transparent organizational climates while creating opportunities to strengthen interpersonal communication competence among faculty members. Such initiatives may contribute to lower levels of academic burnout and promote healthier and more sustainable academic work environments.

### 6.1 Theoretical and practical implications

This study contributes to the literature by extending understanding of how organizational and personal resources are jointly associated with academic burnout among university faculty. Although previous research has examined the relationships between organizational climate and academic burnout, less attention has been given to the indirect associations linking organizational conditions, interpersonal communication competence, and burnout. By examining interpersonal communication competence within this theoretical framework, the present study provides additional evidence regarding the relationships among these constructs in higher education settings.

The findings indicate that organizational climate was associated with both lower levels of academic burnout and higher levels of interpersonal communication competence. Consistent with previous research (Patterson et al., 2005; Schneider et al., 2013), supportive organizational environments characterized by trust, autonomy, and transparent communication are associated with conditions that facilitate effective interpersonal communication. The findings further suggest that both organizational climate and interpersonal communication competence represent important organizational and personal resources associated with lower levels of academic burnout (Demerouti et al., 2001; Schaufeli and Taris, 2014).

The observed indirect association is consistent with the proposition that organizational and personal resources operate together within the Job Demands–Resources (JD-R) framework and Conservation of Resources (COR) theory (Hobfoll, 2011). Interpersonal communication competence may represent one personal resource involved in the indirect association between organizational climate and academic burnout. Given the cross-sectional design, these findings should be interpreted as theoretically informed indirect associations rather than evidence of causal mediation.

From a practical perspective, the findings suggest that efforts to reduce academic burnout should address both organizational conditions and individual competencies. Universities may benefit from promoting supportive organizational climates through transparent communication, collaborative working environments, and fair administrative practices. In addition, professional development initiatives that strengthen interpersonal communication competence may help faculty members manage interpersonal challenges more effectively and enhance access to social support (Cao et al., 2025; Cohen and Wills, 1985). Together, these approaches may contribute to healthier and more sustainable academic work environments.

### 6.2 Limitations and future research directions

Several limitations should be considered when interpreting the findings of this study. First, the cross-sectional nature of the data does not allow conclusions about causal relationships among organizational climate, interpersonal communication competence, and academic burnout. Although the proposed relationships were derived from established theoretical frameworks, future longitudinal studies are needed to clarify the temporal ordering and causal relationships among these variables.

Second, the study relied on a convenience sample drawn from higher education institutions in the Gainesville, Florida region. As a result, the findings may not fully represent the experiences of faculty members in other institutional or geographical contexts. Replication across different institutional, cultural, and national settings would help determine the extent to which the findings are generalizable.

Third, all variables were measured using self-report instruments. Although procedural steps were taken to minimize common method bias, self-report data may still be influenced by factors such as social desirability and individual response tendencies. Future studies may benefit from incorporating multiple data sources or complementary research designs.

The changing nature of academic work also provides opportunities for future research. The growing use of hybrid work arrangements, digital teaching practices, and technology-mediated communication highlights the potential importance of factors such as technostress and digital workload (Watermeyer et al., 2021; Wray and Kinman, 2022). Examining these factors may provide a more comprehensive understanding of faculty well-being in contemporary higher education.

Finally, qualitative and mixed-methods research may offer a deeper understanding of how faculty members experience organizational climate, develop interpersonal communication competence, and cope with burnout in their everyday professional lives (Gutman et al., 2024).

### 6.3 Recommendations

#### 6.3.1 Enhancement of institutional climate

In demanding academic environments, particularly within research-intensive and regional higher education institutions, fostering a supportive organizational climate may represent an important strategy for promoting faculty well-being. Consistent with Patterson et al. (2005), organizational climates characterized by professional autonomy, organizational integration, and supportive supervisory practices represent valuable organizational resources for faculty members. In addition, equitable workload distribution and transparent administrative practices may support faculty perceptions of fairness and the quality of academic working life (Gutman et al., 2024; Misra et al., 2021).

Within the Job Demands–Resources (JD-R) framework (Demerouti et al., 2001), these organizational practices are consistent with strengthening organizational resources associated with lower levels of academic burnout. Accordingly, institutional initiatives aimed at improving the organizational climate may contribute to healthier, more supportive, and sustainable academic work environments.

#### 6.3.2 Communication-oriented professional development

Given the observed associations between interpersonal communication competence and academic burnout, universities may benefit from providing structured communication-focused professional development opportunities. Drawing on the frameworks of Hargie (2011) and Rubin and Martin (1994), such initiatives may be tailored to the communication demands commonly encountered in academic environments (Cao et al., 2025).

In the context of increasing *publish-or-perish* pressures, these initiatives may support faculty well-being and interpersonal functioning alongside academic productivity. Relevant areas of focus may include conflict management, stress-sensitive communication, and effective interaction under demanding workload conditions.

Beyond formal training programs, institutions may also foster collaborative communication environments that facilitate peer support and constructive professional interactions among faculty members. In this context, interpersonal communication competence may be viewed not only as an individual characteristic but also as a personal resource that can be strengthened through organizational support and professional development. Strengthening this resource may help faculty members build supportive workplace relationships and may be associated with lower levels of academic burnout (Cao et al., 2024; Cao et al., 2025).

#### 6.3.3 Well-being-oriented institutional practices

The findings of this study highlight faculty well-being as an important component of healthy and sustainable academic work environments. Within higher education, increasing performance expectations and workload demands may contribute to chronic occupational stress (Shin and Jung, 2014; Turner and Garvis, 2023). Institutions may therefore benefit from adopting practices that balance performance expectations with employee well-being.

Examples of such practices include supportive leadership, transparent communication processes, accessible support systems, and initiatives that promote psychological well-being. Integrating well-being considerations into institutional practices may strengthen supportive organizational conditions and improve faculty members’ workplace experiences (Cao et al., 2025; Dewangan and Goswami, 2025). These recommendations are consistent with previous research emphasizing the importance of supportive organizational environments for employee well-being and lower levels of academic burnout (Cao et al., 2025; Wang and Kee, 2025).

Overall, institutional efforts that promote both organizational effectiveness and faculty well-being may contribute to healthier and more sustainable academic work environments.

## Data Availability

The raw data supporting the conclusions of this article will be made available by the authors, without undue reservation.
